# Effects of Preterm Birth on Intrinsic Fluctuations in Neonatal Cerebral Activity Examined Using Optical Imaging

**DOI:** 10.1371/journal.pone.0067432

**Published:** 2013-06-28

**Authors:** Yutaka Fuchino, Nozomi Naoi, Minoru Shibata, Fusako Niwa, Masahiko Kawai, Yukuo Konishi, Kazuo Okanoya, Masako Myowa-Yamakoshi

**Affiliations:** 1 Japan Science Technology Agency, Exploratory Research for Advanced Technology (ERATO), Okanoya Emotional Information Project, Honcho, Kawaguchi, Saitama, Japan; 2 Graduate School of Education, Kyoto University, Yoshida-Honmachi, Sakyo, Kyoto, Japan; 3 Graduate School of Medicine, Kyoto University, Kawaharacho, Shogoin, Sakyo, Kyoto, Japan; 4 Center for Baby Science, Doshisha University, Kizugawa, Kyoto, Japan; 5 Graduate School of Arts and Sciences, The University of Tokyo, Komaba, Meguro, Tokyo, Japan; 6 RIKEN Brain Science Institute, Wako, Saitama, Japan; Institute of Psychology, Chinese Academy of Sciences, China

## Abstract

Medical advancements in neonatology have significantly increased the number of high-risk preterm survivors. However, recent long-term follow-up studies have suggested that preterm infants are at risk for behavioral, educational, and emotional problems. Although clear relationships have been demonstrated between preterm infants and developmental problems during childhood and adolescence, less is known about the early indications of these problems. Recently, numerous studies on resting-state functional connectivity (RSFC) have demonstrated temporal correlations of activity between spatially remote cortical regions not only in healthy adults but also in neuropathological disorders and early childhood development. In order to compare RSFC of the cerebral cortex between preterm infants at term-equivalent ages and full-term neonates without any anatomical abnormality risk during natural sleep, we used an optical topography system, which is a recently developed extension of near-infrared spectroscopy. We clarified the presence of RSFC in both preterm infants and full-term neonates and showed differences between these groups. The principal differences were that on comparison of RSFC between the bilateral temporal regions, and bilateral parietal regions, RSFC was enhanced in preterm infants compared with full-term neonates; whereas on comparison of RSFC between the left temporal and left parietal regions, RSFC was enhanced in full-term neonates compared with preterm infants. We also demonstrated a difference between the groups in developmental changes of RSFC related to postmenstrual age. Most importantly, these findings suggested that preterm infants and full-term neonates follow different developmental trajectories during the perinatal period because of differences in perinatal experiences and physiological and structural development.

## Introduction

Medical advancements in neonatology have significantly increased the number of high-risk preterm survivors [1]–[5]. However, recent long-term follow-up studies have suggested that preterm infants without apparent brain injuries are at risk for behavioral, educational, and emotional problems [2], [6] and are more likely to meet the criteria for developmental disorders such as attention-deficit hyperactivity disorder (ADHD) [7], pervasive developmental disorders [8], learning disabilities [9], and psychiatric disorders such as anxiety disorders and depression [10]. Although clear relationships have been demonstrated between preterm infants and developmental problems that occur later in childhood and adolescence, little is known about the early indications of these problems.

Supekar et al. (2010) reported that functional and structural maturation of neural networks that comprise distinct regions is an important feature of brain development. Recently, numerous studies on resting-state functional connectivity (RSFC) have demonstrated temporal correlations of activity between spatially remote cortical regions in adults that were assessed by blood oxygen level-dependent signals in functional magnetic resonance imaging (MRI). These fluctuations, which are thought to reflect the presence of intrinsic functional connectivity, have been shown across various distinct networks that support different cognitive functions, including motor regions [11], the anterior cingulate cortex [12], striatum [13], amygdala [14], precuneus [15], insula and cingulate cortex [16], thalamus [17], and insula [18].

RSFC studies have been conducted not only in adults but also in children with developmental disorders such as ADHD and autism spectrum disorder (ASD). The default mode network (DMN) in youth with ADHD have atypical connectivity patterns [19] and atypical functional connectivity between the dorsal anterior cingulate cortex and DMN compared with age-matched controls [20]. Furthermore, adolescents and young adults with ASD have decreased interhemispheric functional connectivity [21] and reduced RSFC in the anterior and posterior regions of the insula compared with age-matched controls [22]. Children with ASD also exhibited prominent patterns of ectopic striatal circuits [23]. These studies highlighted the differences in RSFC between children with developmental disorders and typically developing children and indicated that RSFC analysis can be an early screening tool for developmental disorders.

However, RSFC does not always remain stable throughout an individual’s lifetime, indicating possible developmental changes of RSFC. Recently, RSFC examinations have been performed to assess cerebral cortex development [24]–[33]. These studies have shown the existence of RSFC in humans of all ages, even in preterm neonates [33]–[35]. They also have shown developmental changes of RSFC with regionally specific developmental trajectories of varying complexity levels [34], [35]. As development progresses, cortical activity becomes more coherent across recognized neuroanatomical systems with a general increase in interhemispheric correlations. These studies have indicated that RSFC changes over an individual’s lifetime and there is a possibility that developmental changes of RSFC are affected by synapse formation, pruning, myelination, and experience (learning mechanisms) [24], [36]. Moreover, RSFC has been examined in the developing cerebral cortex to examine differences between preterm infants at term-equivalent ages and full-term neonates using MRI [34], [35]. Smyser et al. (2010) reported that connections between the thalamus and sensorimotor cortex and the brainstem and cerebellum were significantly stronger in full-term control infants than in preterm infants at term-equivalent ages. However, Doria et al. (2010) showed no significant difference between preterm infants at term-equivalent ages and full-term neonates, but they observed a similar effect. Smyser et al. (2010) found that these differences were as a result of neuropathological alterations and postnatal experiences. The differences in the conclusions of these two studies may be explained by differences in neuropathological evidence, postnatal experiences, the use of sedation, and/or postmenstrual age (PMA).

We evaluated the differences in RSFC between preterm infants at term-equivalent ages and full-term neonates without sedation or injuries by assessing RSFC during natural sleep over the same age interval relative to conception (matched PMA). If developmental changes in RSFC over this period were determined solely by intrinsic maturation processes (independent of time in utero or neonatal experience), we would expect to find similar RSFC patterns in preterm and full-term infants of equal PMA. On the other hand, if cortical development is affected by preterm birth, we would expect to find differences in RSFC at the same PMA and possibly also differences in developmental changes over the same PMA range.

## Methods

### Participants

A total of 49 infants [25 females and 24 males; average gestational age (GA): 35.4 weeks, range: 23.1–41.3] participated in the present study (Table 1). They were divided into 2 groups on the basis of their GAs. The full-term group (GA ≧ 37 weeks) comprised 15 females and 9 males (average GA: 39.1 weeks, range: 37–41.3), whereas the preterm group (GA <37 weeks) comprised 10 females and 15 males (average GA: 31.8 weeks, range: 23.1–36.7). The preterm group included 6 extremely low-birth-weight infants (<1000 g). The full-term and preterm groups did not include any infants with anatomical abnormality risks using MRI analysis at term-equivalent.

**Table 1:** Demographic Data for infants.

	Preterm		full-term		*t*-value	*p*-value
	average	range	average	range		
GA (weeks)	31.8	23.1–36.7	39.1	37–41.3	−9.8	<10^−12^
CA at scan (days)	52.2	4–129	4.7	2–8	7.7	<10^−9^
PMA at scan (weeks)	39.3	37.1–42.1	39.8	37.5–42.1	−1.4	0.16
head circumference (cm)	33.9	30.1–37.1	33.3	31.1–37	1.3	0.20
time window for analysis (s)	233.4	150.1–380.3	204.2	150.1–310.3	1.8	0.07

GA: gestational age, CA: chronological age, and PMA: postmenstrual age.

### Ethics

This study was approved by the Ethics Committee of Kyoto University Hospital, Kyoto, Japan (E581). The infants’ caregivers were not paid. Written informed consent was obtained from all caregivers and all were made aware that they could withdraw their children from the study at any time.

### Data Acquisition

**Figure 1:**
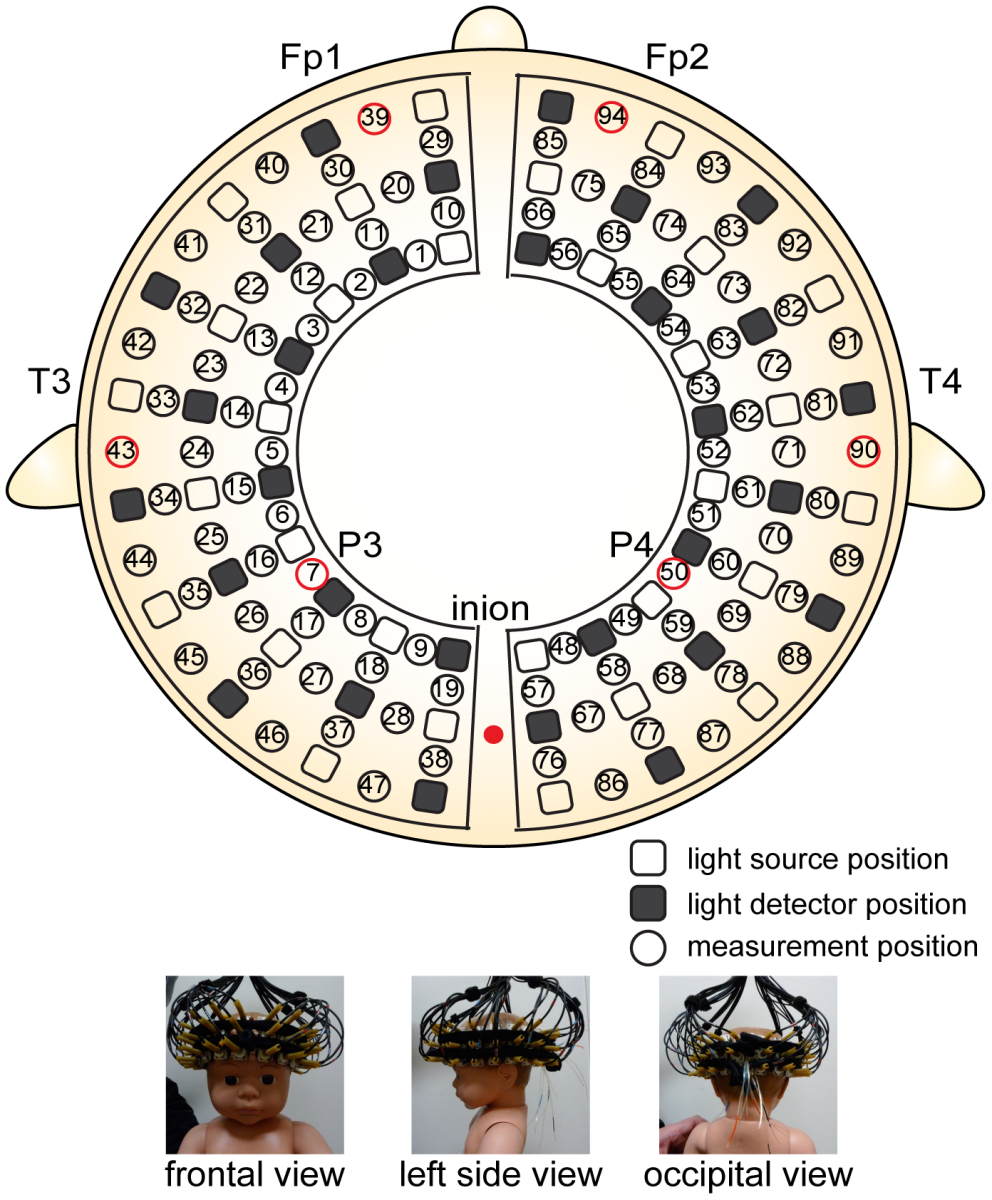
Arrangement of measurement positions. The lowest lines of the probes correspond to the T3-Fp1-Fp2-T4 line according to International 10–20 electrode system. The vertical midline of the channels was centered in the nasion–inion line, and the channels between the 5th and 6th probe in the lowest line corresponded to T3 on the left and T4 on the right.

We used an optical topography (OT) system with 94 measurement channels (ETG-7000, Hitachi Medical Corporation, Tokyo, Japan) for data acquisition. The ETG-7000 is FDA approved. The 2 light sources were near-infrared laser diodes with wavelengths of 785 and 830 nm. The maximum power of each light source was 0.4 mW and the laser power was class 1 M; therefore, the incident light presented no danger of photodynamic damage to skin, bone, or cortex. The reflected light was detected by avalanche photodiodes that were positioned approximately 20 mm from the emitters. Although an array of detector–emitter pairs with a 3-cm distance has been typically used to obtain spatial patterns of cortical activation in adults [37], a detector–emitter distance of approximately 2 cm provides better sensitivity to cortical responses in infants [38] and in Monte Carlo simulation of neonates [39]. The signals at the 94 positions were measured by configuring 30 irradiation positions and 30 detection positions, as shown in Figure 1. All 94 signals were measured and saved simultaneously at a sampling interval of 100 ms.

The probe holders of the OT system were placed on the infant’s bilateral frontal, temporal, parietal and occipital regions (Figure 1). The lowest measurement position line in the frontal region was positioned along the Fp1–Fp2 line in accordance with the international 10–20 system for electroencephalography. The center of the lowest measurement position line in the temporal region was adjusted to coincide with the T3 and T4 scalp sites. The center of probe holder in the occipital region was adjusted to coincide with the infant’s inion.

All infants were held by a pediatrician for measurements during natural sleep within 2 h of feeding. The acquisition time was 3–6.5 min. OT recording ended when the infant awoke or began crying but was continued for up to 6.5 min if the infant remained asleep and relatively motionless. OT recording was performed in a quiet room in the maternity unit. The infant’s behavior was recorded on a check sheet and digital video camera (HDR-XR520V, Sony Corporation, Tokyo, Japan) during the measurement to remove data corrupted by motion artifacts for post-signal preprocessing.

### Signal Preprocessing

The signal preprocessing analysis, which focused on relative changes in the concentration of oxygenated hemoglobin (Δ[oxy-Hb]), was performed using the modified Beer–Lambert law [37]. The Δ[oxy-Hb] data were analyzed using the plug-in-based analysis software Platform for Optical Topography Analysis Tools (freely available at http://goo.gl/4Nqgo, in Japanese) based on Matlab (The MathWorks, Inc., Natick, MA, USA). We passed the data through a bandpass filter set between 0.01 and 0.08 Hz, a widely used range in fMRI-based RSFC studies although there are differences between in frequencies of RSFC used in fMRI studies and in that of NIRS studies and differences in frequencies of RSFC between different functional systems in human adult [40], [41]. We removed data corrupted by artifacts, sudden jumps and slowly-varied U- or inverted-U-shaped spikes, that originated from rapid movements, such as Moro reflexes and spontaneous smiles, which was confirmed by the automated detection based on positive correlation between Δ[oxy-Hb] and relative changes in the concentration of deoxygenated hemoglobin [42] and visual inspection. The excluded data was defined as data from the artifact peaks before 20 s to that after 30 s.

### Correlation Analysis

We calculated the correlation coefficients (r) between the time course of a single channel and the time course of all other measurement channels for each infant data set. (_94_C_2_ = 4371 pairs). Next, Fisher’s *z*-transformation was applied to improve the normality of the correlation coefficients [*z* (*r*)].

### Statistical Analysis

To determine the functional connectivity within the measurement groups (preterm infants and full-term neonates), one-sample *t*-tests were performed to see if the *z* (*r*) values for each channel were significantly different from zero. The calculated *t* values were then converted to *z* statistics according to *z* = (*t* −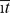)/σ, where 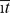 and σ are the expected value and standard deviation, respectively. Results were displayed utilizing a *z* threshold of 0 to determine the brain regions that showed significant connectivity to a single channel.

First, we evaluated the differences in RSFC between the preterm group and the full-term group at the same PMA, and then examined developmental changes in RSFC within each group over the same PMA range.

For comparing the functional connectivity between groups, the *z* (*r*) values were analyzed by two-sample *t*-tests for each measurement channel. The calculated *t* values were then converted to *z* statistics. The measurement channel threshold was set at z >3.29 (corresponding to *p*<0.0005; uncorrected).

In addition, we evaluated the developmental changes in functional connectivity by calculating the correlation coefficients between z(r) values and PMA at the time of the scan. The displayed results fulfilled the criteria of significance (*p*<0.005; uncorrected).

## Results

### Demographic Data

We evaluated the differences in GA, chronological age (CA), and PMA at the time of the scan between the preterm and full-term groups (Table 1). There were significant differences in GA (*t* = −9.8, *p*<10^−12^) and CA at the time of the scan (*t*  = 7.7, *p*<10^−9^) between the preterm and full-term groups. However, there was no significant difference in PMA at the time of the scan (*t* = −1.4, *p*  = 0.16) between the preterm and full-term groups. We evaluated the differences in head circumference and found no significant difference between the preterm and full-term groups (*t*  = 1.3, *p*  = 0.20). In addition, we evaluated the differences in time window for analysis and found no significant difference between the preterm and full-term groups (*t*  = 1.8, *p*  = 0.07).

### The Probability of the Motion Artifacts Appearing

We compared the distribution of motion artifacts for all infants in the preterm and full-term groups. In the preterm group, no motion artifact was observed during NIRS recording in 12 of 25 infants (48%), whereas the recordings from 2 preterm infants (8%) each exhibited 1 artifact, 8 recordings (32%) had two artifacts, 2 recordings (8%) had 3 artifacts, and 1 (4%) had 4 artifacts. In the full-term group, no motion artifact was observed in 9 of 24 infants (37.5%) during NIRS recording, whereas 6 full-term infants (25%) showed one artifact during NIRS acquisition, 7 infants (29.2%) exhibited 2, and 2 full-term infants (8.3%) had 3 artifacts during recording. There was no significant difference in the number of motion artifacts between groups (*t* = −0.11, *p*  = 0.91).

### RSFC in Preterm Infants and Full-term Neonates

**Figure 2:**
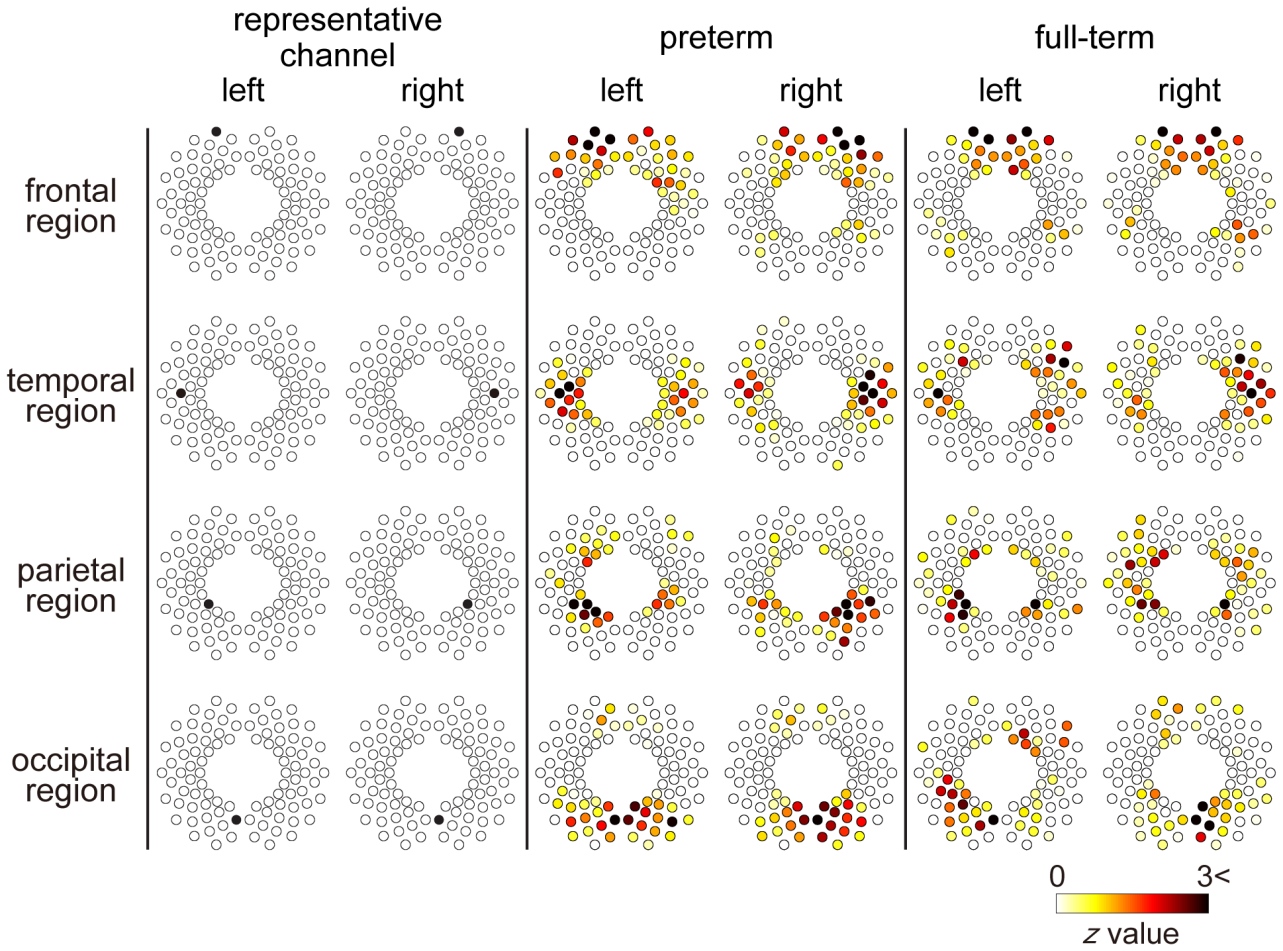
Representative correlation maps corresponding to various measurement channels. Results are displayed utilizing a *z* threshold of 0 in order to determine the brain regions that showed significant connectivity to a single channel. These networks were identified by examining representative channels that were located in the frontal, temporal, parietal, and occipital regions. We also evaluated the spatial distribution of RSFC in all other channels (Figure S1 and S2). Preterm: preterm infant group, full-term: full-term neonate group.

We evaluated the spatial RSFC distributions in the preterm and full-term groups. These networks were identified by examining representative channels that were located in the frontal, temporal, parietal, and occipital regions (Figure 2). Each RSFC was identified in the preterm and full-term groups. Interhemispheric connections between homotopic regions were identified in both groups (Figure 2). We also evaluated the spatial distribution of RSFC in all other channels (Figure S1 and S2). Almost all channels in the frontal, temporal, parietal and occipital regions showed interhemispheric connections between homotopic regions in both groups.

### Comparison of RSFC in Preterm and Full-term Groups

**Figure 3:**
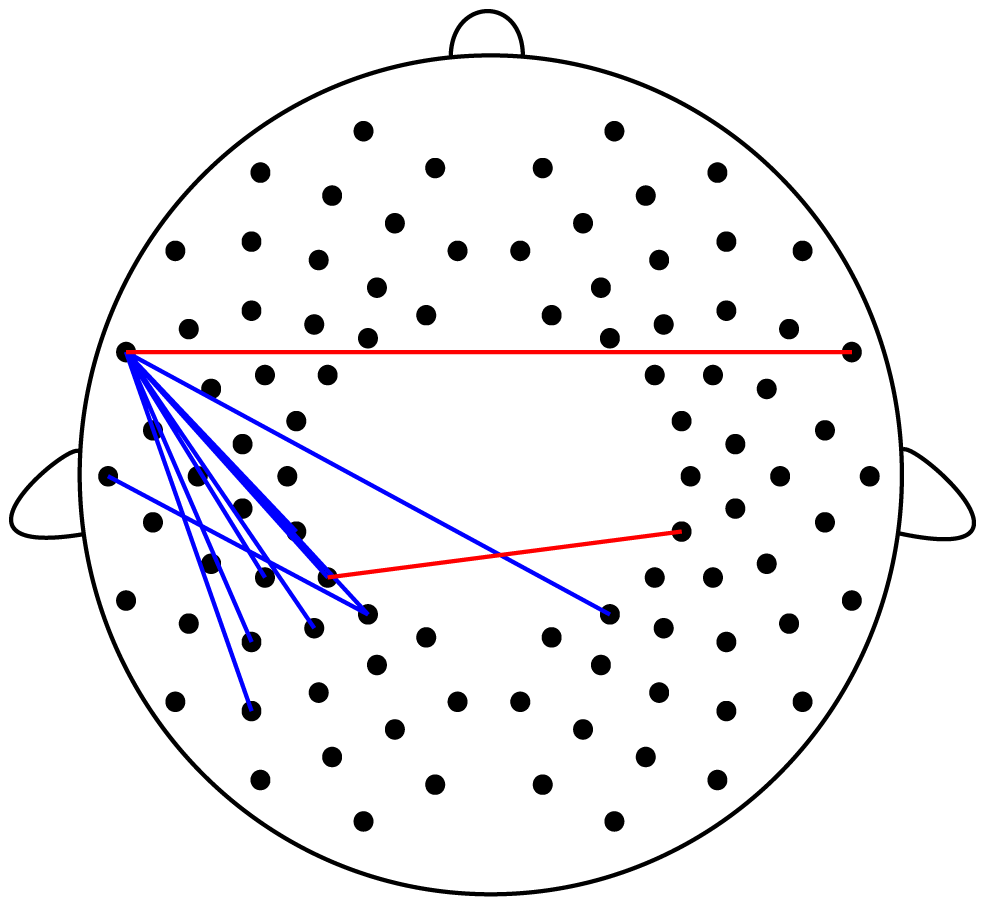
Significant differences in functional connectivity between preterm infants at term-equivalent ages and full-term neonates. The lines represent *z* values >3.29 (corresponding to *p*<0.0005, uncorrected). The red lines show the connections significantly higher in preterm infants. The blue lines show the connections significantly higher in full-term neonates.

To clarify the differences between the networks in the preterm and full-term groups, we computed the differences between RSFC of each channel (Figure 3). Figure 3 shows the measurement positions of the significant channel pairs (*z* >3.29, corresponding to *p*<0.0005, uncorrected). On comparing RSFC in the following regions, the preterm group showed enhanced RSFC compared with the full-term group: the bilateral temporal regions (42―91ch) and the bilateral parietal regions (7―51ch). On comparing RSFC between the left temporal and left parietal regions, the full-term group showed enhanced RSFC compared with the preterm group (42―6, 7, 8, 16, 17, 26, 36ch, and 43-8ch).

### Quantification of Developmental Changes of RSFC in the Preterm and Full-term Groups

**Figure 4:**
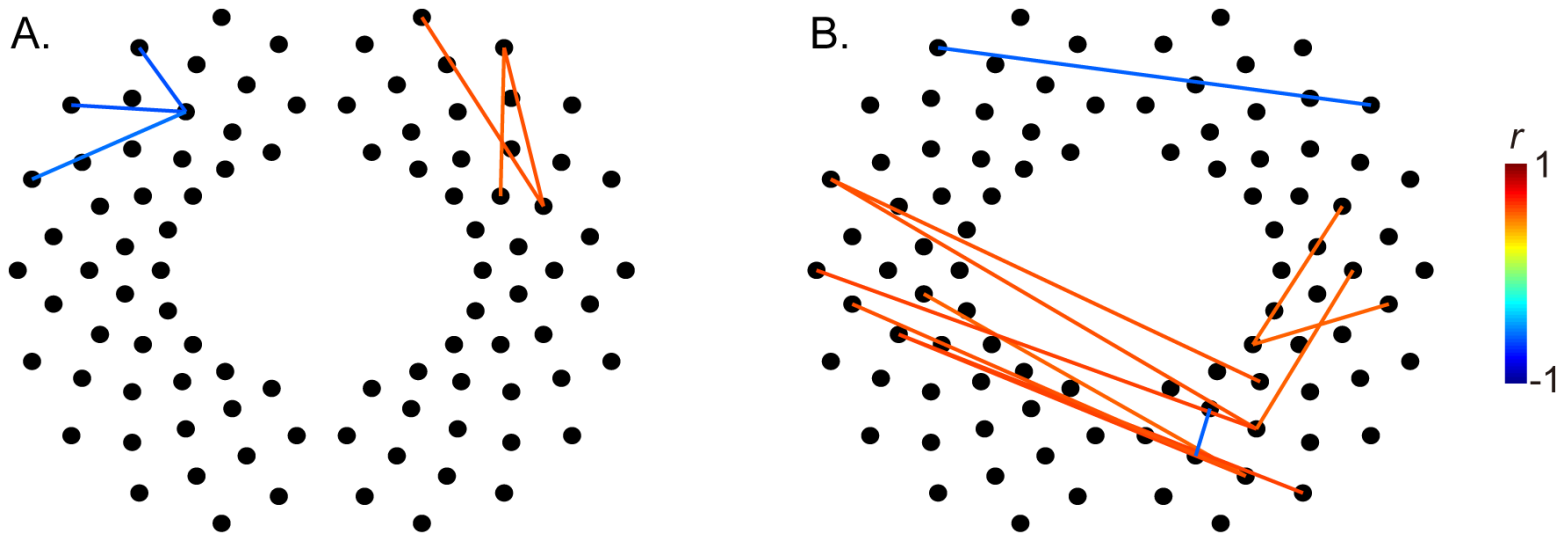
Developmental changes of functional connections. Each line showed correlation coefficients (*r* values) between *z* (*r*) values in measurement channels for each channel and PMA at the time of the scan in the preterm (A) and full-term groups (B). The displayed results fulfilled the criteria of significance (*p*<0.005, uncorrected). Detailed results of the relationship between each line are shown in Supplementary Figures S3 and S4.

To clarify the developmental changes of RSFC, we calculated the correlation coefficients between *z* (*r*) values in measurement channels and PMA at the time of the scan (Figure 4 and details presented in Figures S3 and S4).

In the preterm group, the left prefrontal networks showed significant decreases with PMA and right prefrontal networks showed significant increases with PMA (Figure 4A). Although the left temporal, right parietal, and occipital networks showed significant increases with PMA in the full-term group, the bilateral frontal networks showed significant decreases with PMA (Figure 4B).

## Discussion

The present OT study revealed the presence of RSFC in preterm infants at term-equivalent ages and full-term neonates and detected several differences in RSFC between these groups. The principal differences were that on comparing the bilateral temporal regions, and bilateral parietal regions, RSFC was enhanced in preterm infants compared with full-term neonates. Moreover, on comparing the left temporal and left parietal regions, RSFC was enhanced in full-term neonates compared with preterm infants. We also demonstrated the differences in developmental changes of RSFC related to PMA on comparing the preterm and full-term groups. These results suggested that preterm infants and full-term neonates follow different developmental trajectories during the perinatal period because of differences in perinatal experiences and physiological and structural development.

### RSFC in Preterm Infants at Term-equivalent Ages and Full-term Neonates

In our study, RSFC in full-term neonates was identified and almost all channels in the frontal, temporal, parietal and occipital regions showed interhemispheric connections between homotopic regions (Figures 2 and S2). These results were in agreement with previous MRI studies [34], [35] and OT studies [27], [33], such that, interhemispheric connections between homotopic regions were found in the frontal [27], [35], temporal [34], and occipital regions [33]–[35]. In addition, we evaluated RSFC in preterm infants at term equivalent ages, which also indicated that almost all channels showed interhemispheric connections between homotopic regions, similar to the full-term group (Figures 2 and S1).

We evaluated the differences between the networks in the preterm and full-term groups (Figure 3). Full-term neonates exhibited enhanced RSFC in the left temporal and left parietal regions. A recent diffusion tensor MR imaging (DTI) study of white matter growth in normal human infants reported sparse myelination of the corpus callosum (CC) and arcuate fasciculus at birth and rapid myelination during the first postnatal year [43]. Geng’s study also showed leftward asymmetric growth patterns of arcuate fasciculus tracts in the first year. Arcuate fasciculus tracts are considered to be related to language function [44], a left-dominant function in most humans. We suggest that longer postnatal auditory exposure in preterm infants may accelerate development of these tracts, although future studies are necessary to confirm this proposal.

In addition, a previous study in full-term healthy infants using NIRS revealed RSFC between left temporal and parietal areas in neonates and bilateral RSFC between temporal areas in 3-month-old infants (Homae et al., 2010). Homae et al., also found that RSFC in the parietal area changed from unilateral to bilateral during the first 3 postnatal months. These developmental changes in full-term healthy infants are in accord with our results, i.e., the preterm group exhibited greater bilateral connectivity than full-term infants at a mean PMA of approximately 39−40 weeks, which corresponded to a significant difference in CA (52.2 days vs. 4.7 days; Table 1) and again suggests accelerated formation of these pathways with longer postnatal experience. For example, preterm infants would possibly have far more postnatal exposure to high frequency sounds and language at a time when full-term infants are still in utero (and experience only highly filtered acoustic stimuli).

We also demonstrated differences in developmental changes of RSFC related to PMA between the preterm and full-term groups over the same age interval relative to conception (matched PMA) (Figure 4, S3, and S4). There was no significant difference in mean PMA at the time of the scan (t = −1.4, p  = 0.16; Table 1), PMA ranged approximately more than 1 month (37–42 weeks) in both groups, allowing for an analysis of developmental changes in RSFC. Preterm infants have a shorter gestational age (GA) and a longer chronological age (CA) than full-term infants at the same PMA (PMA = GA+CA) and we demonstrated multicollinear relationships between GA and CA. These relationships remained significant in multiple regression analysis. Therefore, we evaluated the correlation between the RSFC strength and PMA at time of the scan in our study.

### Effects of Experiences during Perinatal Period

Preterm infants and full-term neonates have different developmental experiences during the prenatal and postnatal periods. In utero, exogenous sounds may have an effect on fetal central nervous system development [45]. Although sounds in the environment of a mother’s womb penetrate the tissues and fluids surrounding the fetal head, exogenous low-frequency sounds penetrate the uterus without reduction in sound pressure; therefore, attenuation increases with frequency at a rate of approximately 6 dB/octave over the frequency range of 0.125–2.0 KHz. The same is true of the visual system. There is a difference in the visual system between the intrauterine and extrauterine environments. Measurements performed in pregnant rats and guinea pigs have demonstrated that only 2% of the incoming light of wavelengths below 550 nm was transmitted in utero, and this value increased with increase in the wavelengths of the signal to reach 12% around 650 nm [46]. In a study of sheep, maximal intrauterine light values were observed at noon, which corresponded to 4.7% of incident light transmitted in utero [47].

Auditory evoked potentials [48] and visually evoked responses [49], detected by magnetoencephalography, have been recorded in human fetuses around the beginning of the third trimester. Because preterm infants are born earlier than full-term infants, preterm infants have more sensory experiences with various stimuli outside the uterus, including visual and auditory stimuli, than full-term neonates. There were significant differences in GA and CA between preterm infants and full-term neonates in our study (Table 1), which may have affected the observed RSFC developmental differences.

### Anatomical Differences

Although preterm infants without any anatomical abnormality risk were examined in our study, several studies have reported anatomical differences between preterm infants at term equivalent ages and full-term neonates, including white matter abnormalities such as reductions in white and gray matter volumes and ventricular enlargement [50]–[53]. Thompson et al. (2007) reported region-specific reductions (mean difference; from approximately 8% reduction to approximately 30% reduction) in brain volumes in preterm infants compared with term controls. Furthermore, these differences continue to childhood, and preterm birth is affected by reductions in brain volume, surface area, and cortical thickness [54]. Such anatomical differences may have influenced RSFC at early developmental stages in our study. Several studies have indicated that developmental changes of RSFC were related to the structural maturation of neural networks. However, changes in synaptic density and myelination cannot be fully responsible for these observed developmental changes [55]. Developmental changes in intrinsic factors (e.g., the endocrine system) may also have contributed to RSFC development during the perinatal period. Recent investigations have shown a depolarization–hyperpolarization shift of inhibitory neurons in the early developmental stages [56] and hormone-induced transient changes of inhibitory neurons during the perinatal period [57]. Not only extrinsic stimuli (environmental experiences) but also intrinsic factors (the endocrine system and the immune system) may contribute to RSFC. Through longitudinal studies in the future, we intend to clarify the relationship between multiple factors and developmental changes of RSFC.

### Limitations of the Study

The reflected light was detected by avalanche photodiodes positioned approximately 20 mm from the emitters. Although an array of detector–emitter pairs with a distance of 3 cm between them has been typically used to obtain spatial patterns of cortical activation in adults [37], a detector–emitter distance of approximately 2 cm provides better sensitivity to cortical responses in infants [38] and in Monte Carlo simulation of neonates [39]. We used the same OT method as that used by Naoi et al. (2013), which detected the stimulus-dependent hemodynamic responses in the brain to speech sounds [58]. These results demonstrated that the OT signals, measuring a detector–emitter distance of approximately 2 cm, reflect the hemodynamic responses in the cerebrum in infants.

However, we could not accurately compare the anatomical position for each measurement channel and could not consider the anatomical organization for individuals. The probe holders of the OT system were positioned in accordance with the international 10–20 system. We evaluated the differences in head circumference and found no significant difference between the preterm and full-term groups (*t*  = 1.3, *p*  = 0.20). There were differences of a few millimeters in the measurement positions for each group. Therefore, the activation regions may have been underestimated because the activity was evaluated from a rather random dispersion of measurement positions among the infants.

### Future Studies

We suggested that preterm and full-term infants exhibit significantly different developmental trajectories during the early postnatal period. Moreover, because we did not include any infants with gross anatomical abnormalities as revealed by MRI, we propose that these differences in RSFC reflect differences in developmental trajectories altered by reduced gestation time and/or differences in sensory experiences in utero and/or during the longer postnatal period. It is possible that this early divergence in cortical development may result in delayed cognitive or emotional difficulties in children born preterm. Further follow-up studies are required to evaluate the relationship between RSFC at term and later development to define possible prodromal signs of clinical importance.

### Conclusion

We evaluated RSFC in preterm infants at term-equivalent ages and full-term neonates using OT and clarified the differences in RSFC between the groups. These results suggested that preterm infants and full-term neonates follow different developmental trajectories during the perinatal period as a result of differences in prenatal experiences and physiological and structural development. To contribute to the early screening of developmental disorders and/or difficulties in preterm infants, the relationships between RSFC and multiple factors need to be evaluated in future longitudinal studies.

## Supporting Information

Figure S1 **Representative correlation maps corresponding to all measurement channels in preterm infants at term-equivalent ages.** Results are displayed using a *z* threshold of 0 to determine the brain regions that showed significant connectivity to a single channel.
(TIF)

Figure S2 **Representative correlation maps corresponding to all measurement channels in full-term neonates.** Results are displayed using a *z* threshold of 0 to determine the brain regions that showed significant connectivity to a single channel.
(TIF)

Figure S3 **Developmental changes of functional connections in the preterm group.** Scatterplots with regression lines show the relation of *z* (*r*) values in measurement channels for each channel to PMA at the time of the scan.
(TIF)

Figure S4 **Developmental changes of functional connections in the full-term group.** Scatterplots with regression lines show the relation of *z* (*r*) values in measurement channels for each channel to PMA at the time of the scan.
(TIF)
