# Supplementary figures and images for: Effects of Preterm Birth on Intrinsic Fluctuations in Neonatal Cerebral Activity Examined Using Optical Imaging
Source: PLoS One. 2013 Jun 28;8(6):e67432. doi: 10.1371/journal.pone.0067432 (PMC3696115; doi:10.1371/journal.pone.0067432)

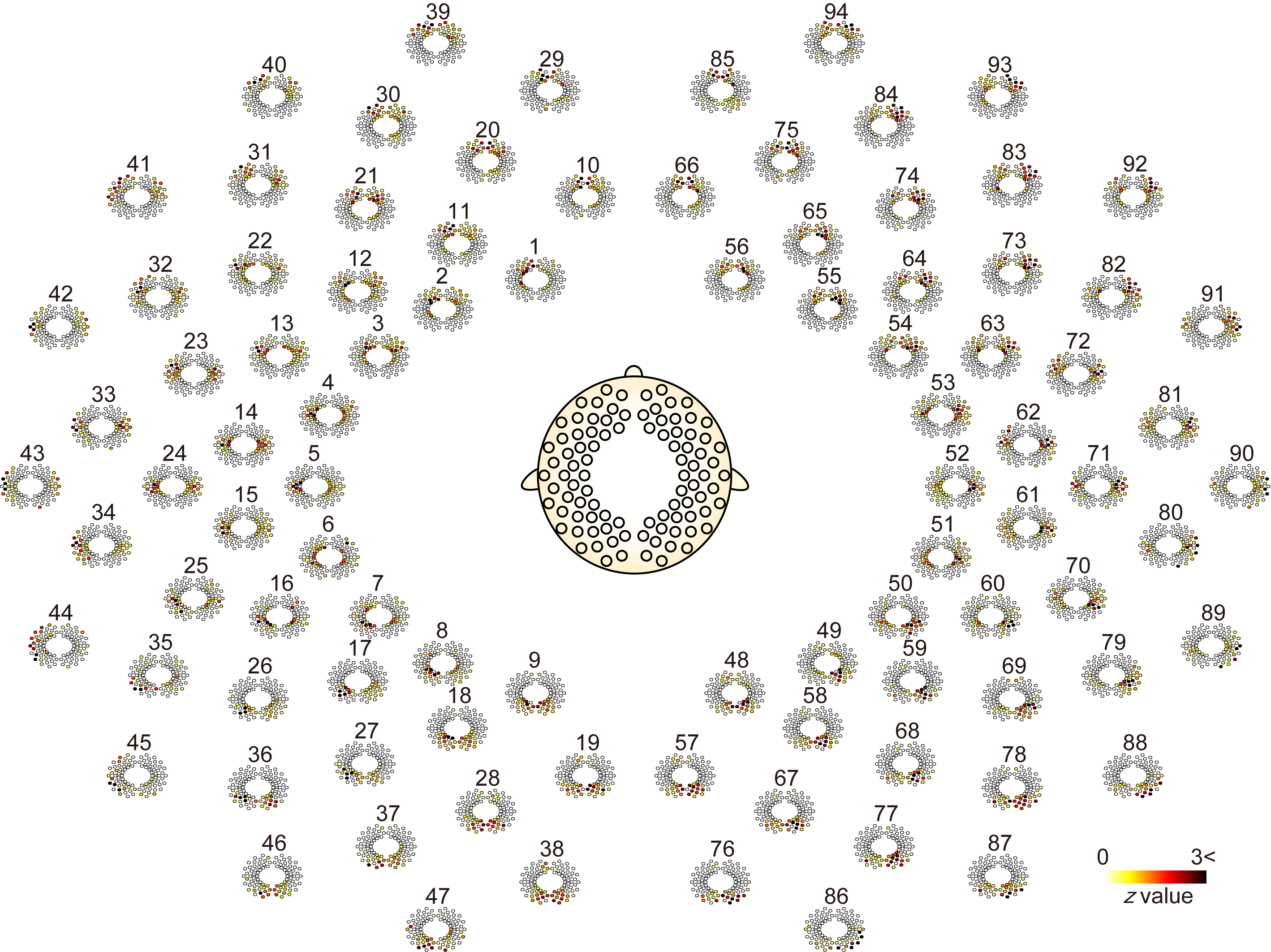

Supplement: Figure S1 — Representative correlation maps corresponding to all measurement channels in preterm infants at term-equivalent ages. Results are displayed using a z threshold of 0 to determine the brain regions that showed significant connectivity to a single channel. (TIF) [file pone.0067432.s001.tif]

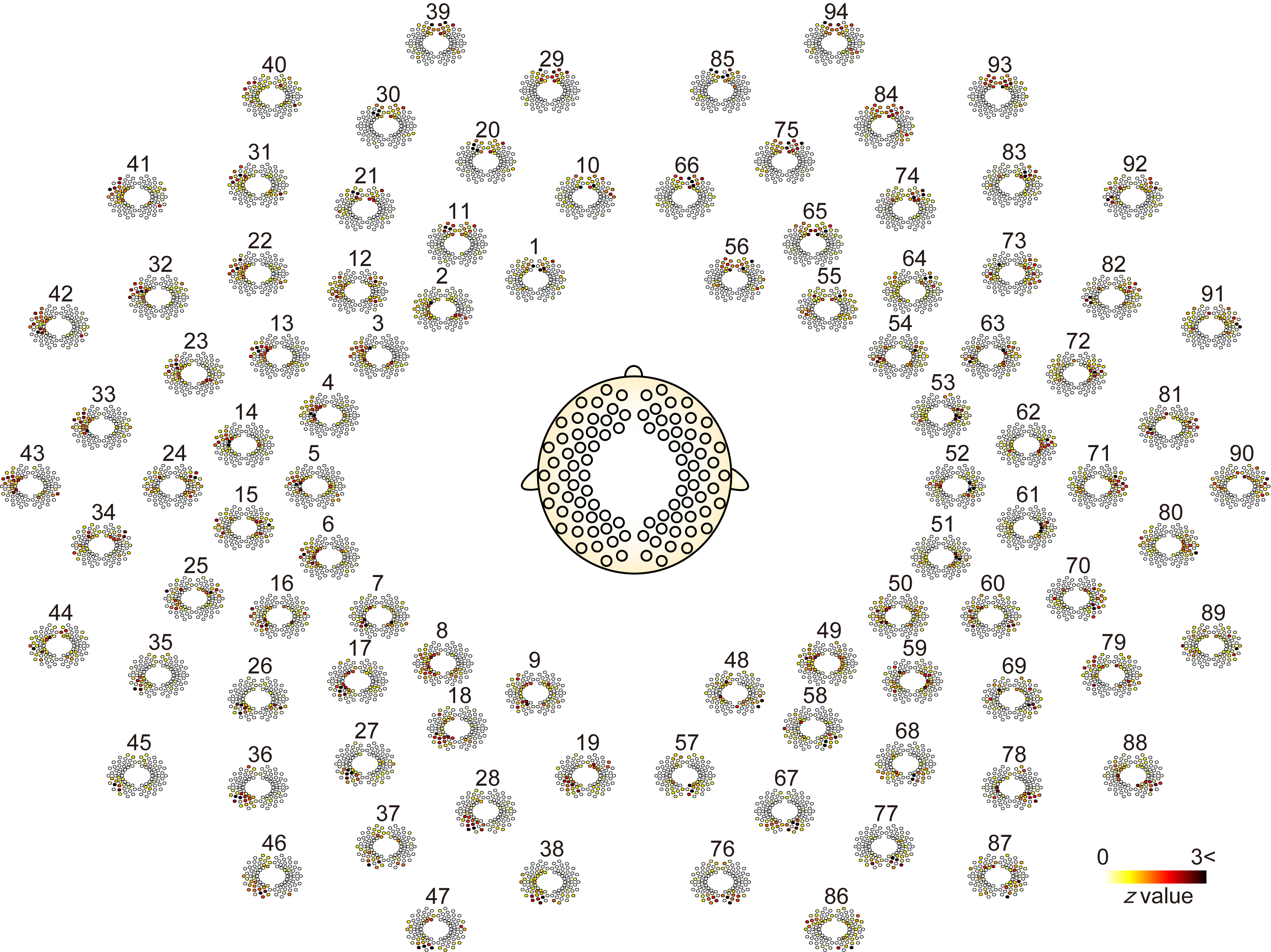

Supplement: Figure S2 — Representative correlation maps corresponding to all measurement channels in full-term neonates. Results are displayed using a z threshold of 0 to determine the brain regions that showed significant connectivity to a single channel. (TIF) [file pone.0067432.s002.tif]

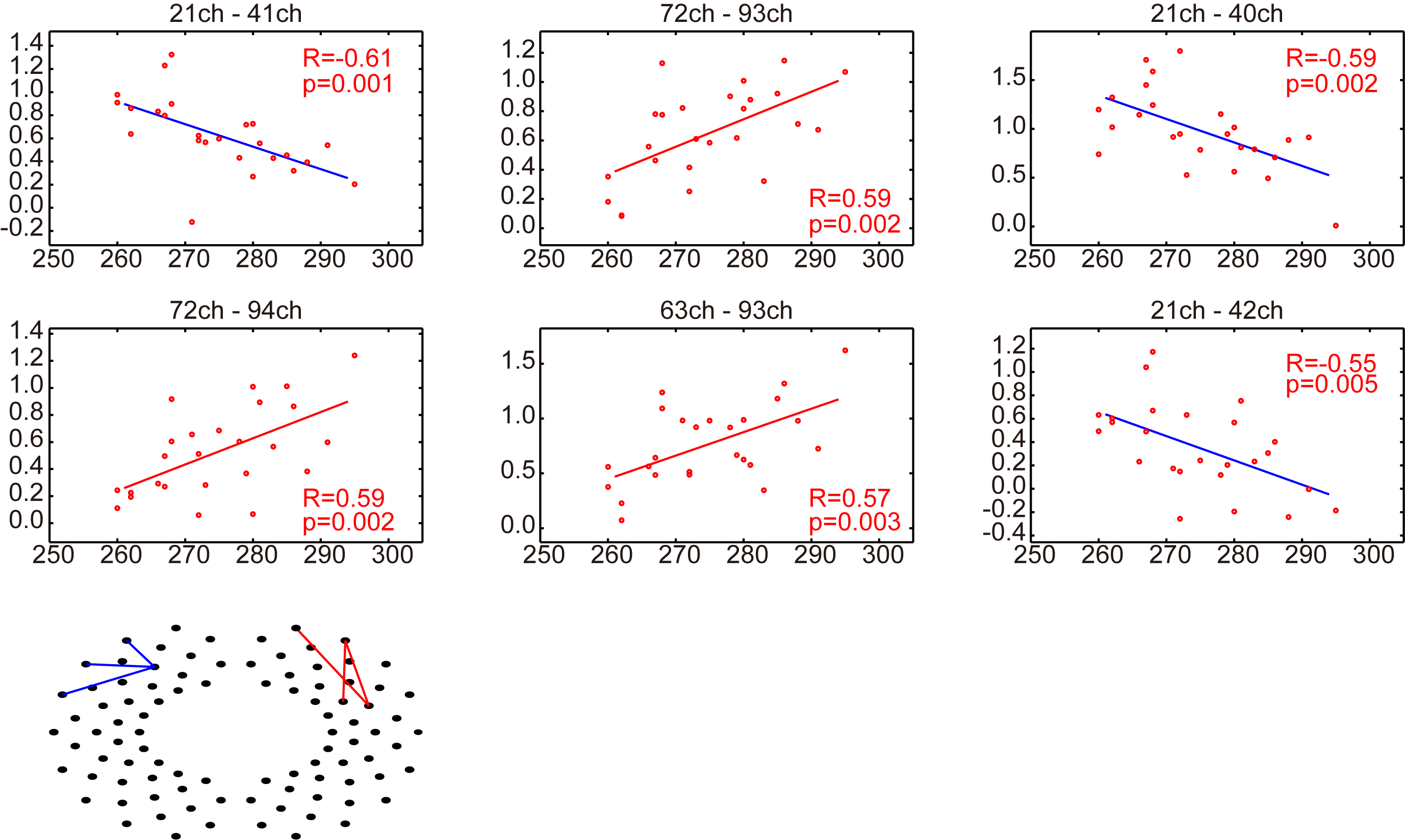

Supplement: Figure S3 — Developmental changes of functional connections in the preterm group. Scatterplots with regression lines show the relation of z (r) values in measurement channels for each channel to PMA at the time of the scan. (TIF) [file pone.0067432.s003.tif]

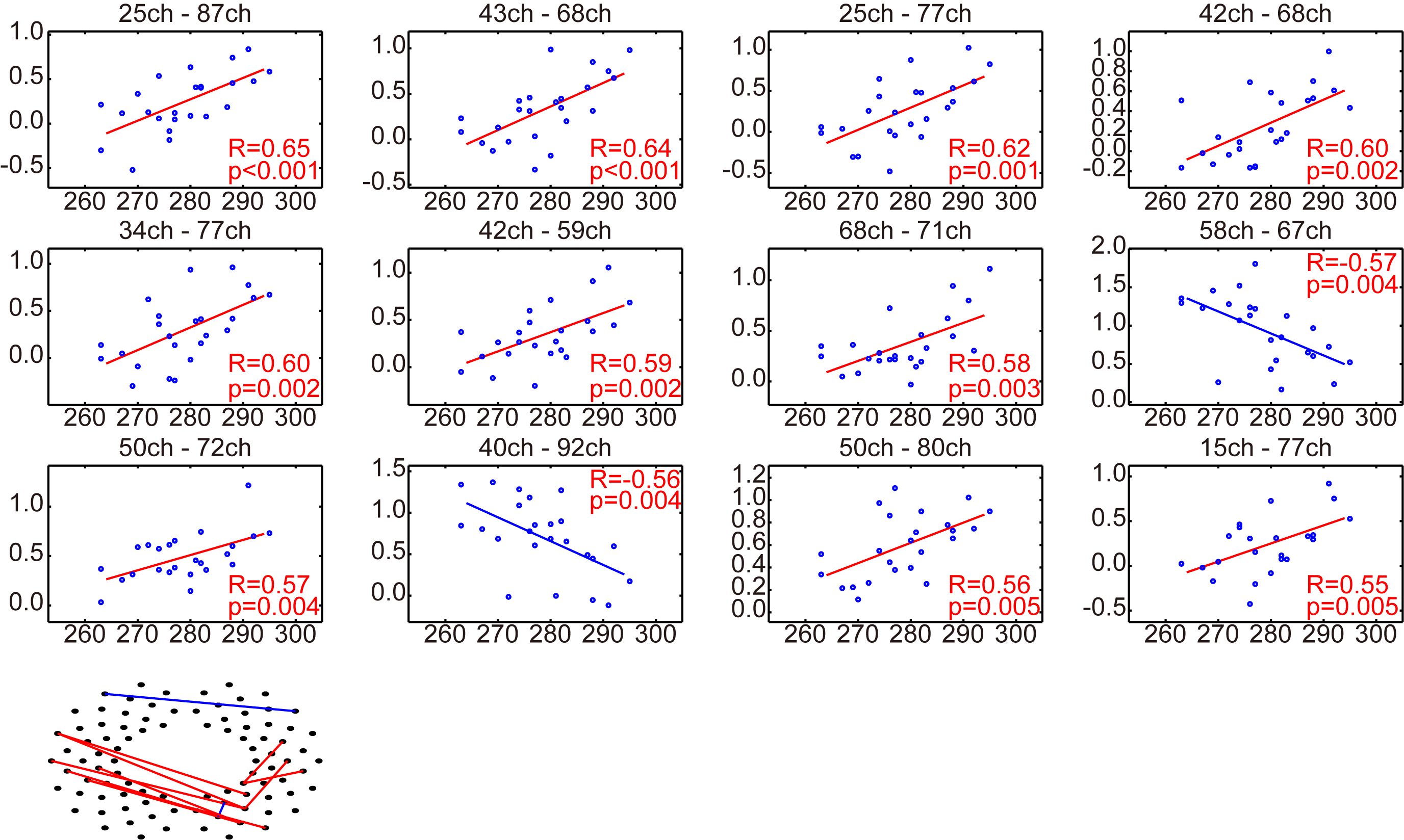

Supplement: Figure S4 — Developmental changes of functional connections in the full-term group. Scatterplots with regression lines show the relation of z (r) values in measurement channels for each channel to PMA at the time of the scan. (TIF) [file pone.0067432.s004.tif]
